# Record of Births and Deaths

**Published:** 1889-10

**Authors:** 


### RECORD OF BIRTHS AND DEATHS.

We are glad to note that through the efforts of our efficient coroner, Dr. J. C. Avary, there has been introduced into the Legislature a bill requiring the registration of all births and deaths. This is a very important measure, to which the JOURNAL will give its heartiest support. Such a law would go far toward limiting the nefarious practices of abortion and infanticide, and is absolutely essential in keeping a correct record of vital statistics. We hope the bill will pass, and that the profession throughout the city and State will urge upon the legislators the necessity of such a law.

### ITEMS.

DR. VIRGIL O. HARDON, of this city, has been spending the last few weeks among the hospitals of New York and Boston.

DR. LOGAN M. CRICHTON has gone to New York to take special courses in diseases of the eye, ear and throat.

THE friends of Dr. D. W. Waggoner will regret to learn of his death, which occurred recently at his home near Athens, Ga. Dr. Waggoner graduated from the Atlanta Medical College in the class of '88. He was valedictorian of his class and graduated with distinction.

DR. W. P. BOND, of Lithonia, presented to the Atlanta Medical College, on the 15th instant, a double-headed baby weighing twelve pounds. Two perfectly developed heads were attached to separate spinal columns, which united at their low extremities.

WE published in the September issue of the JOURNAL a call for the purpose of organizing a "Tri-State Medical Association," to which we again wish to call the attention of the profession. The meeting will be held in Chattanooga on the third Tuesday in October, and many interesting papers have been promised. The session will continue two days. All who desire to read papers are requested to notify the Secretary of the Committee, Dr. Frank Trester Smith.

A CASE OF SENILE DENTITION.—Dr. T. M. Greenwood, of Mineral Bluff, Ga., reports a very interesting case of senile dentition. Patient, female, aged seventy-eight years, mother of twenty-one children at full term. Had never taken a dose of medicine from a physician until June of this year, when Dr. Greenwood was called in and found her suffering with an attack of summer diarrhœa, which lasted for about two weeks. Three weeks thereafter she cut two middle upper incisors, then next two, and last two lower incisors. The gums are much swollen, and there is every indication that she will cut a full set of teeth.

---

INSANITY PROCEEDING FROM THE COLON.—Dr. Harold N. Moyer, in a paper read before the Chicago Medical Society, July 15, 1889, gives some interesting facts and conclusions on the above subject. He reports several cases of melancholia and insanity promptly and permanently relieved by copious enemata. In these cases there is usually a history of obstinate constipation extending over a considerable period, with occasional attacks of watery diarrhœa. Dr. Harold condemns the use of drastic cathartics in obstinate constipation, and recommends in their stead copious injections, which relieve the condition without increasing the irritation. He says : "Regarding the treatment of this condition, we incline to the views expressed by von der Kolk, who says : 'All remedies which act as violent irritants of the colon, the so-called drastics, only increase the tendency to stricture; they add to the sensibility of the colon, and the accumulation of blood in it, and cause watery stools, while the hard masses in the upper portion of the large intestine still remain. The disquietude, the excitement, and the uneasy feeling of the patient are thereby increased, but the strength is diminished if these medicines are continued for any length of time; the circulation becomes more and more irregular, the radial pulse becomes small and the limbs cool."—*Journal of the American Medical Association, August 17, 1889.*

### ITEMS.

AMERICAN PUBLIC HEALTH ASSOCIATION. HEALTH EXHIBITION.—The American Public Health Association will hold its next annual meeting at Brooklyn, N. Y., October 22, 23, 24 and 25, 1889.

This Association comprises over eight hundred members, all devoted officially or otherwise to its declared purpose—the advancement of sanitary science and the promotion of organizations and measures for the practical application of public hygiene. In the furtherance of this purpose it has met annually, during the last sixteen years, in different cities of the United States and Canada, and has, in every instance, had the effect of greatly stimulating public effort in the promotion of health and measures for its maintenance.

With the hope of still further magnifying this interest and effort, it is the purpose of this Association, through its local committee, at the forthcoming meeting, to provide an exhibition of everything available adapted to the promotion of health.

Professor A. R. Robinson, of New York, has been appointed by the Committee on Organization of the International Congress of Dermatology and Syphelography, to be held in Paris, to open the discussion on the subject, "lichen."

APPOINTED PROFESSOR OF PATHOLOGY.—At the last meeting of the Faculty of the Medico-Chirurgical College of Philadelphia, Dr. Ernest Laplace was appointed Professor of Pathology. Professor Laplace is a native of New Orleans, and a graduate of the Literary Department of the Georgetown University, D. C. After several years' study in Tulane University and the Charity Hospital, he went abroad and graduated in the "Faculté de Medicine de Paris," where he studied under Pasteur and Cornil. He afterward spent seven months in Vienna under Billroth and Strickler, and a year in Berlin with Koch and Von Bergmann. While with Koch he discovered the superior efficiency as a germicide of acid sublimate of mercury, and the sulpho-carbolic acid as a disinfectant. After his return to New Orleans he instituted a Pasteur Laboratory for the treatment of Hydrophobia, and was elected visiting surgeon to the Charity Hospital. At the time of his call to the city he was Professor of Physiology and Hygiene in the High School Department of the Tulane University, and Demonstrator of Microscopical Anatomy and Bacteriology in the Medical Department of the same school.

Dr. Samuel Wolfe, of Skippack, Pa., will fill the chair of Physiology for the coming year.

ON THE SO-CALLED TOXIC ANTAGONISM OF SOME POISONS.—Besides the physiological antagonism of poisonous substances, researches have been made by some authors to find a toxic antagonism or an antidotal role of two poisons taken *vis-a-vis* in the organism. Atropine and morphine have been regarded as toxic antagonists.

M. G. H. Roger ("Jour. des soc. sci.," No. 21, 1888) has experimented on rabbits, with the purpose of determining the existence or non-existence of a toxic antagonism between hydrochloride of morphine, neutral sulphate of atropine, sulphate of quinine and potassium chloride. To be enabled to draw experimental conclusions, the author determined the poisonous dose of each element, or the toxic equivalent of Bouchard. (This dose is the toxic amount of the substance necessary to be dissolved in 20 c. c. of distilled water.) The intravenous injections were made with a rapidity of 4 c. c. a minute to the kilogramme of the animal's weight. After M. Roger's experiments with mixed injections of morphine and potassium chloride, each substance acted, as regarded its toxic equivalent, as if it had been injected alone. Often both poisons acted synergetically, and their corresponding toxic effects were added to each other (morphine and atropine; quinine and morphine; atropine and quinine). In some cases the toxicity of the mixture was decidedly greater than the sum of the poisonous effects which should be expected to exist (quinine and potassium chloride). In the course of exploration of the substances named, the author has never succeeded in finding a toxic antagonism, a more or less complete neutralization of one poison by another.—*N. Y. Medical Journal.*

THE AMOUNT OF ALBUMEN IN DIET.—Medical men are in favor of the use of animal food in moderate quantity. But it is very difficult, physiologically speaking, to say what is a moderate quantity, and most men eat as much flesh meat as they feel inclined for without any consideration of the kind. Another attempt to estimate accurately the albumen actually required for tissue-change has been made recently in the chemical laboratory of the Berlin Pathological Institute by Dr. Kumagawa, a native of Japan. Five series of experiments were made upon himself with the following diets—namely, one series with the usual Berlin diet, two with a mixed Japanese diet, and two with a purely vegetable diet—boiled rice, The results of feeding on the last diet are given in the *Centralb. f. d. med. Wiss.*, No. 12, 1889. The experimenter's age was 27, his weight 48 kilogrammes; and the problem was to ascertain the minimum albumen supply requisite to keep up that weight, other proximate foods being afforded in due quantity. Previous experiments showed that for this a diet sufficed which corresponded to 2,300 calorics, and which contained from 70 to 90 grammes of albumen per diem. The rice diet afforded 2,500 calorics, but contained very little albumen, of which the daily supply was 50.5 grammes, of carbohydrates 93.7 per cent. On this diet, there was actually a gain of albumen in the body of 5.884 grammes in nine days—that is, 173 grammes of flesh—and the body-weight increased 0.4 kilos. Thus it is shown that an adult may, upon a diet, having less albumen than is destroyed in the fasting condition, not only preserve intact the nitrogenous equivalent (as also Hirschfeld's experiments have proved), but may even increase the albumen deposited as flesh, if only the diet afford sufficient caloric.—*British Medical Journal.*

SUPRAPUBIC LITHOTOMY IN YOUNG CHILDREN.—Dr. Alfred Lendon, Lecturer on Forensic Medicine, University of Adelaide, has operated or vesical calculus in male children under five years of age three times within twelve months, and with complete success. In each case the rectum was distended with the bulb of a spray-producer as a substitute for Petersen's bag, and the bladder washed out and then distended with boracic acid lotion. The skin-incision in no case extended one inch and a half. The bladder was not sutured; the skin wound was brought together with horsehair, and a drainage tube was inserted, which projected into the bladder. The first patient was four years of age; the calculus weighed fifty-eight grains; urine passed through the urethra on the eleventh day, and ceased to pass through the wound on the thirteenth. The second patient was not quite two years old; the stone weighed one hundred grains; the urine passed naturally at the end of the first week, ceasing next day to pass through the wound. In the third case the patient was three and one-half years old; the calculus weighed forty-five grains; the urine passed through the urethra on the seventh day, and ceased to pass through the wound on the nineteenth. The drainage tube was removed on the day of operation in the second and youngest case; on the second day in the first, and on the third day in the third case. Dr. Lendon, who writes his experiences in the *Australasian Medical Gazette*, states that, had the proper instruments been available, he would have performed litholapaxy in preference to lithotomy. For a surgeon who had no special experience of vesical surgery, suprapubic appeared in many respects preferable to perineal lithotomy. In Dr. Lendon's cases the chief difficulty was the extraction of the stone after the opening of the bladder.—*British Medical Journal.*

TREATMENT OF ACNE VULGARIS.—The treatment recommended by Dr. Isaac, of Berlin, consists chiefly in producing sufficiently intense peeling of the skin to remove all obstructions from the sebaceous glands, and eventually also to reduce excessive vascularisation of the skin. This is most effectually achieved by the application of naphthol or resorcin paste.

*Formula.*—℞. Naphthol 1 part, sulpher præcip. 5 parts, sapon virid. and vaseline āā 2 parts—M. f. pasta.—To remain on for half to one hour and to be repeated daily until the skin peels thoroughly which usually requires a few days. In case of a too intense reaction, a 2 per cent. salicylic acid paste or powder readily gives relief. Another formula warmly recommended is the following:—℞. Pulv. cret. alb. 5 parts, *B* naphthol, camphor and vaselin flav. āā 10 parts, sapon. virid. 15 parts, sulpher præcip., 50 parts. M. f. pasta. This preparation should not be applied for longer than a quarter of an hour. Resorcin was used in about 50 cases with most satisfactory results. It is also applied as a soft paste. ℞. Resorcin, zinc. oxyd., amyl āā 5 parts, vaselin flav. 10 parts. M. f. pasta mollis. Isaac has found it advisable to commence with a less concentrated (10 per cent.) ointment, gradually increasing the strength if necessary.

It is important to bear in mind that acne is often merely a symptom of a disturbance of the inner organs, as it is more than likely that the sebaceous glands which become inflamed by the exit of superfluous quantities of bromine and iodine also serve for the removal of other irritating bodies from the organism. In this way the effect of cheese, beer and coffee on the skin is satisfactorily explained, and the necessity of avoiding such irritants sufficiently proved.—*Brit. Journ. of Dermat., May, 1889.*

TREATMENT OF ACUTE ECZEMA.—In Kaposi's lectures and other good text books detailed directions are given concerning the treatment of the different stages and varieties of eczema, which leave nothing to be desired. Yet there is no disease so often maltreated as acute eczema. Instead of directing the patient not to wash himself, taking care to remove all sources of irritation, and using dusting powder to shield the skin from the effects of heat, sweat and friction, a mixture perhaps containing arsenic is given. The patient is supplied with an ointment which may have been recommended by some authority for chronic eczema. If any directions be given about its application, they are so vague that the friends of the patient are left to do with the ointment what they please. Instead of treating an individual skin, an abstract disease is prescribed for. The effect of this may be very disastrous in the case of children, as the following example shows. A child about eighteen months old, who had been under treatment for more than a year, presented, when I first saw it, a most pitiable object. The entire face and forehead was raw and bleeding, the skin in the bends of the elbows and knees and between the thighs was in the same condition. The scalp was coated with crusts, the buttocks were covered with raised, red, moist, coalescent patches, and on the trunk were numerous red raw-looking papules and groups of papules. The child was very irritable, especially during the night, and for fourteen months the wretched parents were deprived of their night's rest. She had been bathed twice a day, orthodox ointments and lotions innumerable had been tried in vain, and latterly quack remedies were proved to the mother's satisfaction to make the disease rapidly worse. About a month after water was forbidden, under the application of glycerine of borax and glycerine, of each 1 drachm, lanolin to 10 drachms with zinc and starch powder, the skin was completely healed.

Mackintosh (*The Practitioner*, July, 1889) says that "the patient must wash neither with nor without soap, nor with bran-water, nor with oatmeal, milk and water, butter-milk, whey, sour milk, or rain water." He gives internally an aperient saline mixture. During the night he applies emollient ointments, and powders the skin during the day. His favorite ointment is—bismuthi subnit. 4 drachms, zinci oxidi 1 drachm, acidi carbolica liquidi ½ drachm, vaselini alb. 2 ounces. He describes the following as "a beautiful cooling cream, which acts as a balm to the irritable skin"—℞. Bismuthi subnit. 2 drachms, zinci oxcidi ½ drachm, glycerine (Price's) 1½ drachms, acidi carboli liq. 20 mins., vaselin alb. 6 drachms. For tingling and irritation at night he recommends this lotion—℞. Bismuthi subnit. 1 drachm, glycerine (Price's) 4 drachms, acidi carbol. liq. 12 mins., aquam rosæ ad 1 ounce. S. Shake up and apply with a camel's hair brush. For powder he uses equal parts of crimolite, subnitrate of bismuth and zinc oxide,—*Medical Chronicle*, *Sept.*, 1889.

BARETTE.—"On the treatment of enlarged tonsils."—*Revue de Laryngologie, d'Otologie, et de Rhinologie*, June, 1889.

Considering first the medical treatment adopted with the object of arresting the development of the disease at the onset, the author speaks more or less favorably of the usual remedies employed for this purpose. Such are anti-scrofulous drugs, application of lemon juice, insufflations of powdered alum, douches and inhalations of sulphurous acid solutions, and applications of iodine.

The surgical treatment he divides into two classes—cutting and caustic methods. Evulsion, scarification, crushing, and chemical caustics, are not advocated. Removal by bistoury and amygdalotome are considered, and their drawbacks pointed out, and finally preference is given to cauterization by means of the thermo-cautery or the galvano-cautery. The paper is concluded by a description of the method of employing these means.—*Medical Chronicle*, *Sept.*, 1889.

### TENTH INTERNATIONAL MEDICAL CONGRESS AT BERLIN, 1890.

WE, the undersigned, do hereby give notice that, according to the resolution passed at the Washington meeting, September 9th, 1887, the

TENTH INTERNATIONAL MEDICAL CONGRESS

will be held in BERLIN.

The congress will be opened on the 4th and closed on the 9th day of August, 1890.

Detailed information as to the order of proceedings will be issued after the meeting of the delegates of the German Medical Faculties and Medical Societies at Heidelberg on the 17th of September in the current year.

Meanwhile, we should feel sincerely obliged if you will kindly make this communication known among your medical circles, and add in the same time our cordial invitation to the congress.

VON BERGMANN.
VIRCHOW.
WALDEYER.

OKINTCHITZ (EVGENY S.). "Clinical bacteriology of blood in certain infectious diseases of wounds (septicæmia, phlegmon, and pyæmia.")—*St. Petersburg Inaugural Dissertation*, 1889, pp. 86.

FOLLOWING the suggestion by Professor M. J. Afanasieff, Dr. Okintchitz, of Mlava, has undertaken the bacteriological examination of the blood in surgical cases of septicæmia, phlegmon, and pyæmia. The principal outcome of the researches may be summarized somewhat as follows:—

(*a*) Septicæmia (4 cases, 7 examinations: (1) There exist two essentially different varieties of the disease, one of which is caused by the poisoning of the patient's system with some chemical products of decomposing tissues; such are, for instance, cases of senile or spontaneous gangrene of the foot, etc. (2) The other form is produced by the systemic infection with certain pathogenic microbes. (3) In the former variety—in the septic poisoning—the patient's blood does not contain any microbes, though the latter may be easily detected about primary foci. (4) Meanwhile, in cases of the septic infection, there is found in the blood (during the patient's life) a peculiar microbe, which seems to be indentical with Burdoni-Uffreduzzi's proteus hominis capsulatus (*vide* the *Zeitschrift für Hygiene*, 1887, Vol. IV.). [As a matter of fact, in one of the author's two cases of his second variety, the proteus was found; in the other, Fraenkel's diplococcus and the staphylococcus pyogenes albus.] (5) The two varieties of septicæmia have nothing in common but few clinical symptoms and morbid lesions. In regard to prognosis and treatment, they widely differ: while the septic poisoning justifies a relatively favourable prognosis, and demands mainly appropriate operative means, the septic infection is exceedingly grave, and requires such therapeutic treatment as the internal administration of stimulants, antiseptics, etc. (6) In view of these facts, as well as of a totally different causation, the two forms should cease to bear a common name, and be regarded as quite distinct pathological entities. (7) At all events, the subject fully deserves further extensive and careful researches, based essentially on the bacterioscopic examination of the blood.

(*b*) *Phlegmon* (8 cases, 15 examinations): (1) In cases of phlegmon, the patient's blood sometimes (in 3 out of 8 cases), contains the staphylococcus pyogenes citreus (in 1), or cereus (in 1), or albus (in 1). (2) The microbes, however, can be demonstrated only by means of cultivation experiments, and not by a simple microscopic examination. (3) Their appearance in the blood, seemingly, does not stand in any connection with either the intensity of fever, or daily oscillations of the body temperature. [However, in such cases where they were found, the daily difference between the maximal and minimal standard amounted only to 0.2° to 0.6°C, while in the remaining patients a remittent fever existed.] (4) The only pronounced clinical difference between the mycotic and non-mycotic cases seems to consist in gastric disturbances, which are present in the former variety.

(*c*) *Pyæmia* (3 cases, 6 examinations): (1) The pyæmic blood invariably contains either the streptococcus pyogenes (2 cases), or staphylococcus pyogenes aureus (1), which may be detected mostly (in 4 out of 6 examinations) even by an ordinary microscopical examination. (2) Since the hæmic microbes show any signs of scission but very seldom, comparatively with the bacteria found in primary foci, the supposition is justified that their proliferation takes place mainly in the pus and not in the blood, hence thorough disinfection and destruction of the foci, abscesses, etc., represent a matter of paramount importance. (3) Neither the time of the day nor the daily difference in the temperature do produce any influence on the occurrence of microbes in the blood. (4) A great numerical strength of hæmic microbes seems to have a very bad significance in regard to prognosis, and that even in the absence of any metastases about internal organs. (5) On the contrary, a scanty amount of the microbes justifies a favorable prognosis even in the presence of metastases, provided all other conditions, especially the patient's general state, management of the case, etc., are favourable. (6) The staphylococcus seems to have a tendency to a secondary localization, especially about the joints; the streptococcus in cellular tissue. [Meanwhile, Kransfeld and Pavlovsky lay down a diametrically opposite proposition.] (7) The bacillus pyocyane seems to represent a more or less constant follower of the streptococcus. It appears, however, to belong to saprophytic microbes, and to make its invasion only after the soil has been previously prepared for it by the said pyogenic bacterium.

In conclusion, Dr. Okintchitz emphasizes a great importance of the bacteriological examination of the blood in all cases of disease, since it would powerfully promote our knowledge of causes, diagnosis, prognosis and treatment of all infectious affections.—*Medical Chronicle, Sept. 18, 1889.*

### PROGRAMME OF TRI-STATE MEDICAL ASSOCIATION.

CHATTANOOGA, TENN., September 26, 1889.

DEAR DOCTOR: The following papers have been promised for the meeting of the Tri-State Medical Association:

Demonstrations with the Microscope—Prof. James A. Reeves, Chattanooga.

Stricture—Prof. Daniel H. Howell, Atlanta, Ga.; Dr. F. B. Sloan, Cowan, Tenn.

A case of Typhoid Fever with Subnormal Temperature and Subnormal Pulse—Dr. A. S. Wiltse, Kismet, Tenn.

A Plea for the Medical Education of Females—Dr. Chas. P Gordon, Dalton, Ga.

Choleo-Cystotomy, with a case—Dr. E. E. Kerr, Chattanooga.

Report of a Case—Dr. Wm. T. Blackford, Graysville, Ga.; Dr. W. C. Maples, Bellefonte, Ala.

Physiology of the Heart and its Valves—Dr. W. L. Gahagan, Chattanooga.

Relation of the Specialist to the General Practitioner—Dr. F. W. Skillern, Pikeville, Tenn.

Some points in the Diagnosis of Skin Diseases—Prof. E. A. Cobleigh, Chattanooga.

Imaginary Foreign Bodies in the Throat—Dr. Max Thorner, Cincinnati, Ohio; Dr. J. B. Cowan, Tullahoma, Tenn.

Antiseptic Midwifery—Dr. F. W. McRae, Atlanta, Ga.

Other papers of interest will be presented.

Prof. Robert Battey, of Rome, Ga., has promised to be present.

This meeting will be held in response to the following call issued by a number of societies in Alabama, Georgia and Tennessee:

"The members of the medical profession in Alabama, Georgia and Tennessee are requested to meet in Chattanooga on the third Tuesday in October for the purpose of forming a Tri-State Medical Association. All will be admitted to the meeting of the Association, but the membership will be restricted to graduates of regular medical colleges in good standing."

A constitution will be adopted at this meeting which will regulate all matters pertaining to the society.

The meeting will be called to order at 10:00 A. M., Tuesday, October 15th, at the Chamber of Commerce. The sessions will continue two days.

Fraternally yours,

FRANK TRESTER SMITH, M. D.,
Sec'y of Committee.

